# Effect of nutritional factors on adherence to antiretroviral therapy among HIV-infected adults: a case control study in Northern Ethiopia

**DOI:** 10.1186/1471-2334-13-233

**Published:** 2013-05-23

**Authors:** Negassie Berhe, Desalegn Tegabu, Mekuriaw Alemayehu

**Affiliations:** 1 Department of Public Health Officer, Health Science College, Axum University, Axum, Ethiopia; 2 Department of Health Informatics, Institute of Public Health, College of Medicine and Health Science, University of Gondar, Gondar, Ethiopia; 3 Department of Environmental and Occupational Health and Safety, Institute of Public Health, College of Medicine and Health Sciences, University of Gondar, Gondar, Ethiopia

**Keywords:** HIV/AIDS, Antiretroviral therapy, Nutritional factors, Ethiopia

## Abstract

**Background:**

Adherence to antiretroviral treatment is critical for suppression of viral replication, reduced destruction of CD_4_ cells, prevention of viral resistance, promotion of immune reconstitution and slowed disease progression. This study sought to determine the effect of nutritional factors on adherence to ART among HIV-infected adults on ART.

**Methods:**

Matched case control study design (matched by age and sex) was employed. Data was collected from ART registration chart, pre-tested structured data extraction format, anthropometric measurements and by interview. Conditional logistic regression was used to compute the relevant associations among the variables by STATA version 10.

**Results:**

From 174 paired subjects participated in the study 80 (46%) pair were males and 94 (54%) pair were females on ART for at least one year prior to the survey. The mean age (±SD) for the non-adherent was 38.4 ± 8.1years and for the adherent subjects was 38.5 ± 8.4 years. Malnutrition with BMI less than 18.5 Kg/m^2^ in the adherent group was 14 (8%) and that of the non-adherent group was 74 (42.5%) which was associated with non-adherence to ART (AOR 10.0, 95%CI 4.3 – 54.7). Inability to get enough and quality food was also associated with non-adherence to ART (AOR 2.1, 95%CI 1.1 – 11.5).

**Conclusions:**

Malnutrition, inability to get enough and/or quality food and consumption pattern which is less than three meals per day were significantly associated with non-adherence to ART. Therefore, the capacity to effectively manage the food and nutrition implications of ART adherence is a critical factor in the success of antiretroviral therapy in resource limited settings.

## Background

The Human Immunodeficiency Virus [HIV] pandemic continues to have a pronounced global impact particularly among the world’s resource limited settings [1]. According to Global HIV/AIDS Progress Report of 2011, Globally 34 million people were with HIV at the end of 2010. In sub-Saharan Africa, an estimated 1.9 million people became infected with a total of about 22.6 million in 2010. Access to antiretroviral therapy in low and middle income countries increased to 6.65 million in 2010, 47% coverage of people eligible for treatment. Sub-Saharan Africa had the greatest increase in the number of people receiving antiretroviral therapy to about 5 million in 2010 [1]. In Ethiopia in 2011 adult HIV prevalence was 1.5% [2]. By the end of 2011, a total of 333,434 people had ever started ART. Of those there were 249,174 adults currently on treatment [3].

Adherence to antiretroviral treatment is defined as taking 95% or more of the prescribed doses on time and in the correct way, either with or without food [4]. It is critical for suppression of viral replication, reduced destruction of CD4 cells, prevention of viral resistance, promotion of immune reconstitution, and slowed disease progression [5,6]. However, from those who are on Antiretroviral Therapy [ART], around one out of four ART users fail to achieve optimal adherence due to different reasons like nutritional, social and economic conditions [7]. HIV-positive individuals especially those on ART are prone to malnutrition due to inadequate dietary intake, appetite loss, nutritional losses, metabolic changes, and increased requirements for both macro- and micro-nutrients [8].

Combating under-nutrition and HIV/AIDS are two of the eight United Nations Millennium Development Goals to be achieved by 2015. HIV infection is a global public health emergency and is most prevalent in areas of the world where under-nutrition is also a serious concern [8]. Lack of food security and poor nutritional status may hasten progression to AIDS-related illnesses, undermine adherence and response to antiretroviral therapy and exacerbate socioeconomic impacts of the virus [4]. To prevent such problems high-income countries and international organizations like World Food Program [WFP], World Health Organization [WHO], President’s Emergency Plan for AIDS Relief [PEPFAR], Food and Agriculture Organization [FAO] and United States Agency for International Development [USAID] recommend nutritional support as a part of the care provided to HIV-positive individuals [4,8,9].

In developing countries chronic underlying malnutrition and its intersection with food insecurity, poverty, and co-infections pose a serious threat to efforts to combat HIV/AIDS [10]. Adults living with HIV have 10−30% higher energy requirements than a healthy adult without HIV [4]. High prevalence of HIV, non adherence to antiretroviral [ARV] drugs and malnutrition results in a vicious cycle of under nutrition, poverty, and disease progression [10]. In Ethiopia, approximately 49% of the general population is without adequate nutrition [11]. In addition to this, according to two studies in Ethiopia (Dire Dawa and Jimma zone southwest Ethiopia) the prevalence of food insecurity among People Living with HIV/AIDS [PLHIV] on ART were 90% and 63% respectively [12,13] which indicates adults on ART are suffering from malnutrition.

There are documented evidences which recommend detailed studies on the impact of nutritional factors on adherence to ART [12,14,15]. The effect of nutritional factors on adherence to ART among HIV/AIDS subjects is not well explored, despite the critical role of nutrition in care, disease progression and ART adherence among HIV/AIDS cases. Therefore, the objective of this study is to assess the effect of nutritional factors on adherence to antiretroviral therapy among HIV-infected adults in Tigray region north Ethiopia. The findings of this study could be useful evidence for scholars who are interested in this field, and for the ART and nutrition programs undertaking by government and other non-governmental organizations.

## Methods

The study employed matched case control study design for clients under ART. For the comparison purpose, non-adherent subjects were matched by age and sex with adherent controls.

The study was conducted in four hospitals found in Tigray Regional state. Tigray regional state which is found in north Ethiopia has an estimated total population of 4,314,456 according to Central Statistical Authority [CSA] report in 2007 [16]. It has five zonal Administrative Divisions and there are about 1.8% PLHIV [2], among these about 28,044 are on ART. Tigray Regional state has 14 hospitals and 219 health centers [17]. The ART patients taking treatment and care in ART clinics of the hospitals included in the study are Mekelle hospital 3645, Adwa hospital 876, St. Marry hospital 942 and Suhul hospital 1098. The study period was from August 5/2012 to October 17/2012.

The study population was adult HIV/AIDS positive subjects greater than 18 years who were in ART follow up in four Hospitals of Tigray regional state. Participants with incomplete data for last three visits and women on ART who were on current pregnancy were excluded from the study. Non-adherent participants from document who were adherent in the current visit, and adherent participants with good adherence from document and who were non-adherent at current visit were also excluded.

The sample size is computed by Kelsey formula for case control studies in OpenEpi Version 3.03.17 assuming a ratio of non-adherent cases to adherent controls of 1:1, a two-sided significance level (alpha) of 0.05, Power 80% and assuming 15% difference in nutritional status between the adherent and non-adherent subjects. In a case control study done in Pwani Region eastern Tanzania, the difference between the two groups in educational status, income, occupation and other socioeconomic factors which also are included in our study is less than 12% [18]. However, considering the cost and the design of the study the 15% difference assumption was taken for calculating the sample size. By considering this the sample size calculated for both groups was 171 pair. With adjustment for non-response (5%), the sample size for both groups’ was 180 pair.

From the governmental hospitals found in the Tigray regional state, four hospitals were selected purposely considering the load and the availability of Fluorescence-Activated Cell Sorting [FACS] Count Machine for CD_4_ count and CBC (complete blood count) machines for the purpose of getting an adequate number of non-adherent subjects with better documentation within the given shorter period of data collection. By scanning patient ART follow up charts we selected patients who were non-adherent (poor and fair) at least once in the last three visits for follow up appointment. From the selected non-adherent HIV-infected clients, those who reported having ingested less than 95% of the total number of the prescribed antiretroviral medication for the last one month were considered as “cases”. Age and sex matched adherent controls that were with good adherence in all of the last three visits and those who reported having ingested 95% or more of the total number of the prescribed antiretroviral medication for the last one month was considered as “controls”. Finally data was collected for the matched pair by collecting first the non-adherent cases and then the adherent controls for each case (106 from Mekelle hospital, 28 from Suhul hospital, 21 from St. Marry hospital and 19 from Adwa hospital), until the matched sample size make up the whole sample of 174.

The primary dependent variable was Adherence to ART and other independent variables were defined as a categorical variable with the following:-

*Adherence to ART:* - is defined as taking one’s medicine as prescribed and agreed between the patient and provider which is 95% or more adherence to ART. Which means taking doses no more than two hours before or two hours after the time of a doctor’s advice to take doses (95% or more adherence = missing ≤2 doses of 30 doses or ≤3 doses of 60 doses) [11,19].

*Non-adherence to ART:* - is the condition of missing doses completely, not following information given by a physician, as well as taking drugs inappropriately. Which means taking doses two or more hours before, and/or two or more hours after the time of a doctor’s advice to take doses or missing doses completely (less than 95% adherence = missing >2 doses of 30 doses or >3 doses of 60 doses) [11,19].

*PHQ-9 score for depression (points):* - A score of 1 to 4 - No depression, 5 to 9 - Mild depression, 10 to 14 - Moderate depression, 15 to 19 - Moderately Severe depression and 20 to 27 - Severe depression [20].

*Altitude adjusted hemoglobin*:- the adjustment is subtracted from each individual’s observed hemoglobin level to calculate an *adjusted* hemoglobin. The altitude of Adwa, Shire, Mekelle and Axum is 1882, 1953, 2084 and 2195 meter respectively from sea level which is found in the range 1750≤ m <2250 which will be adjusted by subtracting 0.8 g/dl from the observed hemoglobin [21].

Functional Status: -

•Working actively: Able to perform usual work in or out of the house.

•Ambulatory: Able to perform activities of daily living.

•Bedridden: Not able to perform activities of daily living [22].

In all investigations, the measurement was completed as follows. Height was measured to the nearest 1 centimeter using a portable, free – standing stadiometer. Weight was measured to the nearest 0.01kg using ward or clinical based clinical scales, all of which were calibrated at the start of the study. We were rate good appetite for those study participants who eats full of the plate and/or most of plate and we were rate poor appetite for those study participants who eats half of the plate and less. We have collected data on adequate and/or quality food by using questions of eating pattern and nutritional factors. We were rate adequate for those study participants who get three or more meals per day with and without eating between meals.

Data on demographic factors, nutritional factors, immuno-hematological factors and self report adherence to ART were collected by document review, anthropometric measurements, and by using a structured pre-tested questionnaire which is developed from different literatures. The structured questionnaire was prepared in English version and translated to Tigrigna, and again back to English to confirm the correctness of the translation, efficiency of questionnaire and for analysis purpose. It had detailed questions on predictor factors like socio-demographic characteristics, psychosocial factors, nutritional factors and ART self reported adherence. Interview was conducted to fill the structured questionnaire in their own respective ART clinics after being oriented as to how to do it by trained data collectors and supervisors. The data collectors were 4 case managers, 4 nurses or health officers and 4 MSc students as supervisors.

After coding the data was entered using Excel, data cleanup and cross-checking was done and it was analyzed by using STATA version 10 by principal investigator using conditional logistic regression (clogit) model for matched case control analysis. The association of each variable with non-adherence was tested using the odds ratio, the 95% confidence interval and p value; a p-value less than 0.05 being considered statistically significant.

Ethical clearance was obtained from University of Gondar research and publication office and then the ethical clearance was submitted to Tigray Regional Health Bureau. Official letters given from Tigray Regional Health Bureau was submitted to the respective hospital administration Chief Executive Officers and Medical Directors to get formal permission. The purposes and importance of the study was explained and informed consent was secured from each participant. Confidentiality was maintained at the data collection process and data analysis stages. All of the study participants were assured that the data will be anonymous, names or any personal identifiers will not be recorded and that was done according to the agreement. Participant’s involvement in the study was on voluntary basis and those who wish to quit their participation at any stage were informed to do so without any restriction.

## Results

A total of 174 subjects were participated in the present study, of which 80(46%) pair were males and 94 (54%) pair were females on ART for at least one year prior to the survey. The mean age (±SD) was 38.4 ± 8.1years for the non-adherent and 38.5 ± 8.4 years for adherent subjects. Of the participants 71 (40.8%) from the adherent group and 72 (41.4%) from the non-adherent group belongs to the age group 30 – 39 years (Table 1).

**Table 1:** Socio-demographic and economic characteristics of the study participants comparing adherent and non-adherent in Tigray regional state, northern Ethiopia, 2012

**Characteristics of study participants**	**Status of adherence to ART**	
	**Non-adherent to ART number (%)**	**Adherent to ART number (%)**
**Sex**		
**Male**	80(46)	80(46)
**Female**	94(54)	94(54)
**Age**		
**18 - 29**	23(13.2)	23(13.2)
**30 - 39**	72(41.4)	71(40.8)
**40 - 49**	67(38.5)	66(38)
**≥50**	12(6.9)	14(8)
**Permanent address**		
**Urban**	156(89.7)	159(91.4)
**Rural**	18(10.3)	15(8.6)
**Marital Status**		
**Single**	38(21.8)	39(22.4)
**Married**	67(38.5)	76(43.7)
**Widowed**	38(21.8)	43(24.7)
**Divorced**	31(17.9)	16(9.2)
**Educational status**		
**No education**	31(17.8)	37(21.3)
**Elementary (1 - 8)**	94(54)	58((33.3)
**Secondary (9 - 12)**	38(21.8)	49(28.2)
**12+**	11(6.4)	30(17.2)
**Occupation**		
**Have no Job**	26(14.9)	44(25.3)
**Government employed**	42(24.2)	46(26.4)
**Self employed/business**	54(31)	36(20.7)
**Daily laborer**	52(29.9)	48(27.6)
**Living With**		
**Alone**	67(38.5)	52(29.9)
**Family**	81(46.6)	84(48.3)
**Parents**	9(5.1)	9(5.1)
**With others**	17(9.8)	29(16.7)
**Average Monthly income (Ethio. Birr)**		
**No income**	25(14.4)	26(14.9)
**≤ 500**	95(54.6)	65(37.4)
**501-999**	24(13.8)	31(17.8)
**≥ 1000**	30(17.2)	52(29.9)
**Distance to hospital (Km)**		
**≤10**	112(64.4)	130(74.7)
**>10**	62(35.6)	44(25.3)

Among the study participants 76 (43.7%) from the adherent group and 67 (38.5%) from the non- adherent group were married while 39 (22.4%) from the adherent group and 38 (21.8%) from the non-adherent group were never married. Assessment of educational status of the study participants showed that 79 (45.4%) from the adherent group and 59 (39.6%) from the non-adherent group attended secondary school or higher education (Table 1).

In the assessment of the average monthly income of the study participants 91 (52.3%) from the adherent group and 120 (69%) from the non-adherent group had monthly income below 27 $ or 500 Ethiopian Birr. From the study participants 84 (48.3%) from the adherent group and 81 (46.6%) from the non-adherent group live with their families. The majority of the study participants; 159 (91.4%) from the adherent group and 156 (89.7%) from the non-adherent group were from urban (Table 1).

At initiation of ART close to 135 (77.6%) from the adherent group and 135 (77.6%) from the non-adherent group had CD_4_ count of less than 200 cells/mm^3^. Eighty seven (50%) participants from the adherent group and 105 (60.3%) from non-adherent group had a BMI less than 18.5 Kg/m^2^. The body mass index of the adherent subjects was increased by a mean of 0.4 Kg/m^2^ in the latest four visits (within the last two years). However; there was no mean increment in BMI in the non-adherent participants (Table 2). The mean increment in weight of the adherent group in the latest four visits was 1.2 Kg but the weight in the non-adherent group was decreased by a mean of 0.1 Kg (Table 2). The mean increment in altitude adjusted hemoglobin in the non-adherent participants was 0.4 mg/dl and that of the adherent group was increased by 0.6 mg/dl. In addition to this, the mean CD_4_ count of the adherent participants was increased by a mean of 38 cells and that of the non-adherent participants was increased by a mean of 8 cells (Table 2).

**Table 2:** The trend of clinical & nutritional status among study participants comparing adherent & non-adherent subjects using mean at each visit in Tigray regional state, northern Ethiopia, 2012

**Characteristics of study participants**	**Status of adherence to ART**	**At initiation of ART**	**1st visit of last three visits**	**2nd visit of last three visits**	**3rd visit of last three visits**	**Current visit**
BMI (Kg/m2)	Adherent	18.5	20.2	20.7	21.0	21.5
	Non-adherent	17.9	19.1	19.3	19.1	19.1
Weight (Kg)	Adherent	50.8	55.5	56.8	57.5	59.1
	Non-adherent	48.1	51.5	51.8	51.4	51.3
Observed HGB	Adherent	12.2	13.8	14.2	14.5	14.5
(mg/dl)	Non-adherent	12.3	13.3	13.6	13.8	13.8
Adjusted HGB by	Adherent	11.4	13.0	13.4	13.7	13.7
altitude (mg/dl)	Non-adherent	11.5	12.5	12.8	13.0	13.0
TLC (10^6^ cells/mm^3^)	Adherent	5.5	5.6	5.6	5.7	5.6
	Non-adherent	5.3	5.4	5.6	5.6	5.7
CD4 (cells/mm^3^)	Adherent	155	408	460	495	522
	Non-adherent	140	282	302	324	304

N.B:- *BMI*, Body Mass Index; *HGB*, Hemoglobin; *TLC*, Total Leukocyte Count; CD4=Cluster of Differentiation 4.

Among the study subjects 123 (70.7%) from adherent group and 113 (64.9%) from non-adherent respondents reported that they are satisfied with ART benefit they obtained. From the non-adherent group 21 (12%) and from the adherent group 1 (0.6%) had moderately severe and severe depression (≥15 points). In the last three months 22 (12.7%) from the non-adherent group and 6 (3.4%) from the adherent group were ambulatory in their functional status (Table 3).

**Table 3:** Psychosocial and health service related factors among study participants comparing adherent and non-adherent using a Chi-Square test in Tigray regional state, northern Ethiopia, 2012

**Characteristics of study participants**	**Status of adherence to ART**		**P-value**
	**Non-adherent to ART Number (%)**	**Adherent to ART Number (%)**	
Current cigarette smoking (within 30 days)			0.431
Yes	16(9.2)	12(6.9)	
No	158(90.8)	162(93.1)	
Ever cigarette smoking after starting ART			0.855
Yes	16(9.2)	17(9.8)	
No	158(90.8)	157(90.2)	
Ever khat chewing after starting ART			0.149
Yes	21(12.1)	13(7.5)	
No	153(87.9)	161(92.5)	
Disclosure of ART use			0.659
Yes	148(85.1)	145(83.3)	
No	26(14.9)	29(16.7)	
Family support for ART use			0.411
Yes	117(75.5)	116(79.5)	
No	38(24.5)	30(20.5)	
Satisfaction by ART benefit			0.251
Yes	113(64.9)	123(70.7)	
No	61(35.1)	51(29.3)	
PHQ-9 score for depression (points)			0.000
0 – 4	53(30.5)	115(66.1)	
5 – 9	72(41.4)	50(28.7)	
10 – 14	28(16.1)	8(4.6)	
15 – 27	21(12)	1(0.6)	
Functional status in last 3 months			0.006
Working actively	152(87.3)	168(96.6)	
Ambulatory	22(12.7)	6(3.4)	

In the bivariate analysis adjusted mean HGB of latest four visits, mean total leukocyte count (TLC) of the latest four visits, ever use of alcohol after starting ART, current alcohol use (within 30 days), and PHQ-9 score for depression are significantly associated with non-adherence to ART (Table 4).

**Table 4:** Bivariate associations of the levels of adherence to ART with behavioral, clinical, food & diet related factors among study participants in Tigray regional state, northern Ethiopia, 2012

**Characteristics of study participants**	**Status of adherence to ART**		**COR (95%CI)**
	**Non-adherent to ART Number (%)**	**Adherent to ART Number (%)**	
Daily eating pattern of last 12 months			
Three meals & above	21(12.1)	17(9.8)	1
Two meals & eating between meals	136(78.2)	140(80.5)	0.8(0.4 – 1.6)
Two meals or less	17(9.8)	17(9.8)	0.8(0.3 – 2.3)
Source of food in the last 12 months			
Purchase (market/grocery)	148(85.1)	144(82.8)	1
Household farm/garden	11(6.3)	15(8.6)	0.7(0.3 – 1.6)
Support from others	15(8.6)	15(8.6)	0.9(0.4 – 2.2)
Adjusted mean HGB of the latest four visits (mg/dl)			
<11.5	29(16.7)	3(1.7)	14(3.3 – 58.8)
≥11.5	145(83.3)	171(98.3)	1
Mean TLC of the latest four visits (10^6^cells/mm^3^)			
<4.0	28(16.1)	11(6.3)	2.7(1.3 – 5.60
≥4.0	146(83.9)	163(93.7)	1
Occupation			
• Have no Job	26(14.9)	44(25.3)	1
• Government employed	42(24.1)	46(26.4)	1.6(0.8 – 3.2)
• Self employed/business	54(31)	36(20.7)	2.7(1.3 – 5.3)
• Daily laborer	52(29.9)	48(27.6)	1.8(1.0 – 3.4)
Duration of treatment (months)			
12 – 36	33(19)	28(16.1)	1
37 – 60	72(41.4)	93(53.5)	0.6(0.4 – 1.2)
≥61	69(39.6)	53(30.4)	1.1(0.6 – 2.0)
Current alcohol consumption (within 30 days)			
Yes	73(42)	11(6.3)	13.4(5.4 – 33.2)
No	101(58)	163(93.7)	1
Ever use of alcohol after starting ART			
Yes	140(80.5)	89(51.1)	4.9(2.7 – 8.9)
No	34(19.5)	85(48.9)	1
PHQ-9 score for depression (Points)			
<5	53(30.5)	115(66.1)	1
≥5	121(69.5)	59(33.9)	5.1(3.0 – 8.9)
Current khat chewing (within 30 days)			
Yes	17(9.8)	7(4)	2.4(1.0 – 5.9)
No	157(90.2)	167(96)	1

N.B:- *TLC*, Total Leukocyte Count; *HGB*, Hemoglobin; *NGO*, Non-Government Organization; *PHQ*, Patient Health Questionnaire.

The multivariate analysis was used to identify characteristics that were predictive of non-adherence to ART. Mean BMI of the latest four visits less than 18.5 Kg/m^2^, unable to get enough and/or quality food, consumption pattern of last 24 hours and mean CD_4_ count of the latest four visits less than 350 cells/mm^3^ are independently associated factors with non-adherence to ART (Table 5).

**Table 5:** Multivariate associations of the levels of adherence to ART among study participants in Tigray regional state, northern Ethiopia, 2012

**Characteristics of study participants**	**Status of adherence to ART**		**COR (95%CI)**	**AOR (95%CI)**
	**Nonadherent\ to ART Number (%)**	**Adherent to ART Number (%)**		
**Mean BMI of latest four visits (Kg/m**^**2**^**)**				
**<18.5**	74(42.5)	14(8)	7.7(3.8 – 15.4)	10.0(4.3 – 54.7)
**≥18.5**	100(57.5)	160(92)	r	r
**Unable to get enough and/or quality food**				
**Yes**	119(68.4)	80(46)	2.6(1.6 – 4.2)	2.1(1.1 – 11.5)
**No**	55(31.6)	94(54)	r	r
**Consumption pattern of last 24 hours**				
**Three meals & above**	124(71.3)	163(93.7)	r	r
**Less than three meals**	50(28.7)	11(6.3)	8.8(3.5 – 22.2)	10.9(1.3 – 81.4)
**Mean CD**_4_**count of latest four visits (cells/mm**^**3**^**)**				
**<350**	118(67.8)	25(15.5)	8.6(4.7 – 15.6)	18.3(6.7 – 102.7)
**≥350**	56(32.2)	147(84.5)	r	r
**Appétit in last 7 days**				
**Good**	89(51.1)	135(77.6)	r	r
**Poor**	85(48.9)	39(22.4)	3.6(2.1 – 6.0)	2.0(0.7 – 6.0)
**I could not afford to eat balanced meals**				
**Yes**	160(92)	136(78.1)	2.7(1.5 – 5.0)	2.0(0.5 – 11.9)
**No**	14(8)	38(21.9)	r	r
**Education**				
**No education**	31(17.8)	37(21.3)	4.5(2.0 – 10.3)	5.6(0.7 – 42.9)
**Elementary (1 - 8)**	94(54)	58(33.3)	2.3(1.0 – 5.6)	1.9(0.2 – 14.9)
**Secondary (9 - 12)**	38(21.8)	49(28.2)	2.6(1.1 – 6.2)	0.6(0.1 – 4.2)
**12+**	11(6.4)	30(17.2)	r	r
**Average Monthly income (Ethio. Birr)**				
**• no income**	25(14.4)	26(14.9)	2.6(1.4 – 4.6)	1.4(0.4 – 5.9)
**• ≤ 500**	94(54)	65(37.4)	1.3(0.6 – 2.6)	1.0(0.2 – 6.6)
**• 501-999**	24(13.8)	31(17.8)	1.7(0.8 – 3.6)	3.0(0.6 – 19.1)
**• ≥ 1000**	31(17.8)	52(29.9)	r	r
**Distance to hospital (Km)**				
**≤10**	112(64.4)	130(74.7)	r	r
**>10**	62(35.6)	44(25.3)	1.6(1.0 – 2.6)	1.9(0.8 – 10.3)

N.B:- *BMI*, Body Mass Index; *CD*_*4*_, Cluster of Differentiation 4.

r:- reference.

## Discussion

This study focused on determining the effect of nutritional factors on adherence to ART among adult people living with HIV taking antiretroviral therapy. As a result, abnormality in nutritional factors, dietary patterns and immuno-hematological status were found significantly associated with non-adherence to ART. This is in line with other studies which reported nutritional factors may have an effect on adherence to ART [12,14]. In addition to this, there are few emerging articles which recommend detailed studies on the effect of nutritional factors and dietary patterns of adherence to ART as these may have an effect on adherence to ART [12,14,15].

As to this study result, malnutrition (which is defined as BMI<18.5 Kg/m^2^) was significantly associated with non-adherence to ART. Malnutrition was about 10 times more frequent in the non-adherent participants than in the adherent counterparts. This is in line with a quasi-experimental study in Zambia [14], and a study from Uganda [15] which reported that better nutritional status could enhance adherence to ART. However, this is not in line with the cross sectional study in Dire Dawa Ethiopia which reported nutritional status had no impact on adherence to ART [12]. This study reported that people with good nutritional status miss doses more than those with poor nutritional status [12]. The possible explanation for this difference may be because of the difference in design of the studies. A randomized clinical trial among wasted adult people living with HIV who receives supplementary food for 14 weeks and not, in Blantyre, Malawi also reported that no differences were seen in ART adherence [23]. The reason for this may be because of the shorter time of supplementation to have an effect on adherence to ART.

In this study, the inability to get enough and/or quality food was about 2.1 times higher in the non-adherent group which shows a significant association with non-adherence to ART. In other studies, increasing and integrating nutritional supplementation which improves access to food into ART programs improve adherence and maximize the benefits of ART therapy [14,24]. In the qualitative study from Uganda, South Africa, Tanzania, and Malawi, hunger during HAART initiation emerged as a leading obstacle to ARV adherence [7]. In addition to this, other studies revealed that lack of food as the main challenge to ART adherence [25-27]. The prospective observational study from central Haiti also found that providing food assistance to individuals with HIV and those under food insecurity improves BMI, food security and adherence to clinic visits [28]. This all shows nutritional factors have effects on adherence to ART.

The consumption pattern which is less than three regular meals per day of last 24 hours was significantly associated with non-adherence to ART. In addition to this, poor appetite and unable to afford balanced meals were associated with non-adherence to ART in the bivariate analysis which is supported by the qualitative study from Uganda [6]. In another qualitative study in northeastern Uganda, consuming only one meal per day and being dependent on caregivers for food were risk factors for ARV non-adherence [29]. In addition to this, in Zambia, the belief that ARVs must be taken with food led individuals to skip doses when they could not access enough to eat [30]. The study from Jimma zone south west Ethiopia reported that food insecurity was high among HAART treated people living with HIV/AIDS even though not showing its effect on adherence. That study reported that significant numbers of PLHIV took less than the mean eating occasions and food diversity [13].

In the multivariate analysis of this study the participants with a mean CD4 count of the latest four visits less than 350 cells/mm^3^ was associated with non-adherence to ART compared to the adherent counterpart. In the study of Nutrition for Healthy Living Cohort (NFHL), lower CD4^+^ cell counts were associated with lower weight. Each 100-cell/mm^3^ decrease in CD4^+^ cell count was associated with a 1.9kg lower weight [31]. In a study from West Africa average BMI and CD4 cell count was 20.7 and 20.5, 217 and 191/mm^3^ respectively, an increase in CD4 cell count was around 1.7 times higher (+ 114 vs. + 68 CD_4_ cells/mm^3^ respectively in support and control groups [32] which shows nutritional status may have an effect on treatment outcomes of HIV/AIDS.

In addition to the negative consequences of personal, socioeconomic and provider related factors on day-to-day ARV adherence in other studies, our study elucidated that malnutrition and dietary factors are risk factors for ART non-adherence, which have been shown to be associated with virologic failure, worse clinical outcomes and mortality in another randomized trial of west African adults [33]. In the pilot intervention study in Zambia, individuals receiving food supplementation with HAART achieved significantly higher ARV adherence than individuals not receiving food supplementation [14]. Another study from Uganda also concluded that malnutrition and food insecurity have emerged as major barriers to the effectiveness of ART programs including adherence to ART [15] which is in line with our study’s result.

The findings of this study should be interpreted with some limitations. Our case control study provides statistical associations of effects of nutritional factors on adherence to ART. This cannot establish whether poor nutrition is a cause or a consequence of non-adherence to ART. In addition to this, the study relies on participants’ selection and the data collected from documents filed by different professionals including Health officers, BSc nurses, and Diploma graduated nurses at the different sites of the study area. The reason for this is the four hospitals have different professionals at their ART clinics. Apart from this the selection of non-adherent subjects may have some limitations. These subjects were selected by combining previous and current adherence level criteria to avoid selection bias. However, for the sake of different reasons the non-adherent subjects may be nutritionally affected ones. In addition to this self report of historical events (recall bias) could have been present. Moreover, the use of ART clinic nurses and ART case managers in the hospitals as data collectors might have introduced an interviewer bias.

## Conclusions

Despite the limitations of our study, through construction of a plausible control group and rigorous analysis of collected data, our finding has elucidated the presence of positive associations of malnutrition and other dietary factors on adherence to ART. In this study malnutrition at least mild malnutrition, lack of food and consumption pattern which is less than three meals per day were significantly associated with non-adherence to ART.

## Competing interest

The authors declare that they have no competing interest.

## Authors’ contributions

NB wrote the proposal of this research. DT, MA revised the proposal and incorporate some comments. NB, DT, MA participated in data collection and analysis. MA wrote the final manuscript. All authors read and approved the final manuscript.

## Pre-publication history

The pre-publication history for this paper can be accessed here:

http://www.biomedcentral.com/1471-2334/13/233/prepub
